# Suicide prevention, public health, and the chiropractic profession: a call to action

**DOI:** 10.1186/s12998-021-00372-7

**Published:** 2021-04-14

**Authors:** Zachary A. Cupler, Clinton J. Daniels, Derek R. Anderson, Michael T. Anderson, Jason G. Napuli, Megan E. Tritt

**Affiliations:** 1 Butler VA Health Care System, Butler, PA USA; 2 Institute for Clinical Research Education, University of Pittsburgh School of Medicine, Pittsburgh, PA USA; 3 VA Puget Sound Health Care System, Tacoma, WA USA; 4 St. Louis VA Health Care System, St. Louis, MO USA

**Keywords:** Suicide prevention, Chiropractic, Public health, Biopsychosocial

## Abstract

**Background:**

Suicide is a major public health concern that has wide-reaching implications on individuals, families, and society. Efforts to respond to a public health concern as a portal-of-entry provider can reduce morbidity and mortality of patients. The objective of this commentary is a call to action to initiate dialogue regarding suicide prevention and the role the chiropractic profession may play.

**Discussion:**

This public health burden requires doctors of chiropractic to realize current strengths and recognize contemporaneous deficiencies in clinical, research, and policy environments. With this better understanding, only then can the chiropractic profession strive to enhance knowledge and promote clinical acumen to target and mitigate suicide risk to better serve the public.

**Conclusion:**

We implore the profession to transition from bystander to actively engaged in the culture of suicide prevention beholden to all aspects of the biopsychosocial healthcare model. The chiropractic profession’s participation in suicide prevention improves the health and wellness of one’s community while also impacting the broader public health arena.

## Introduction

Suicide is a public health crisis and a global problem that can be prevented. In 2016, more than 800,000 deaths worldwide were due to suicide with global crude suicide mortality rate at 10.6 per 100,000 population [1]. In 2018, more than 48,000 people died by suicide in the United States (US) [2]. Concerningly, the rate of suicide among all US adults has increased by 33.3% between 1999 and 2017 [3]. Since 2008, suicide has ranked 10th in the leading causes of death in all ages in the US [2]. Special populations such as military veterans, elderly, adolescents, and females are at increased risk of suicidal behavior [4, 5]. While utilization of mental health services is on the rise, non-psychiatric/mental health providers are far more likely to interact with those who commit suicide within 1 year and even within 1 month of the suicide death relative to inpatient and outpatient mental health services [6, 7]. A chiropractor, considered as a portal-of-entry provider in numerous parts of the world [8, 9], is essential in this national and international public health crisis.

Public health practice is a social and political concept aimed at improving health, prolonging life, and improving the quality of life among whole populations through health promotion, disease prevention, and other forms of health interventions [10, 11]. In general, public health initiatives involve comprehensive approaches to promote the well-being of a community across multiple sectors, including healthcare professions, funders, governments, non-governmental organizations, and other stakeholders [12]. Public health approaches for prevention and intervention are framed in the context of society to the individual level and from primordial to quaternary preventions (Table 1) [13].

**Table 1:** Prevention health measures and corresponding to stages of disease. Adapted from Kisling and Das [13].

Natural history stage	Prevention health measure	Definition	Suicide prevention specific example
Underlying	Primordial prevention	Risk factor reduction targeted towards an entire population through focus on social and environmental conditions	Government policy (i.e. lethal means)
Susceptible	Primary prevention	Measures aimed at a susceptible population or individual prior to the occurrence of disease; healthy individuals	Health promotion Suicide prevention education to college students at risk for suicide attempts. Assessing infrastructure for jumping points in military and veteran health care facilities
Subclinical	Secondary prevention	Early disease detection and its target is healthy-appearing individuals with subclinical forms of the disease; screenings; seeks to prevent the onset of disease	PCP performs routine screening for suicide risk factors Treatment for depression or associated risk factor for self-directed violence Crisis counseling for a community after a suicide
Clinical	Tertiary prevention	Implemented in symptomatic patients and aims to reduce the severity of the disease as well as any associated sequelae; rehabilitation efforts	Crisis counseling Inpatient rehabilitation after a suicidal act
Recovery/disability/death	Quaternary prevention	An action taken to protect individuals from medical interventions (over-medicalization) that are likely to cause more harm than good	Ongoing services for the after-effects and consequences of suicide Resources such as suicide survivor support groups

In the contemporary practice of chiropractic, consideration of the biomedical, psychological, and social factors associated with spinal pain is necessary to complete the clinical picture and understanding to develop a care plan for each patient’s needs and make referrals when necessary [14–16]. Dr. Richard Brown, the World Federation of Chiropractic (WFC) Secretary-General, has called for chiropractors to become EPIC: ***e****vidence-based*, ***p****atient-centered*, ***i****nterprofessional*, and ***c****ollaborative* [17]. To this end, we urge chiropractors to concern themselves with the public health approach to suicide prevention at the societal and individual levels. The purpose of this article is to serve as a call to action for the chiropractic profession to participate in this urgent public health concern.

### Chiropractic practice trends

Chiropractors self-identify, and are perceived by many other health care professionals, as experts in, and best suited to complete, musculoskeletal assessment and triage [18–23]. Patients similarly perceive chiropractic providers as experts in musculoskeletal care, particularly back pain, joint pain, and headaches [24]. A literature review examining reports published between 1965 and 2005 found the prevalence of chiropractic care utilization in the general population varied between 6 and 12% [25]. Additional studies such as the Analyses of the Medical Expenditure Panel Survey indicated a slightly lower prevalence estimate with 5.2% in 2008, however, that same study indicated that those seeking chiropractic care were those with concurrent mental health illnesses [26]. Of 14,025 veterans who served in Operations Enduring Freedom/Iraqi Freedom/New Dawn with at least one visit at a Veterans Affairs chiropractic clinic from 2001 to 2014, 54.2% had been diagnosed with post-traumatic stress disorder [PTSD], 47.6% had a diagnosis associated with depression, 19.8% had a substance use disorder diagnosis, 9.3% had a diagnosis of bipolar disorder, and 0.9% had been diagnosed with schizophrenia [27, 28].

**Fig. 1:**
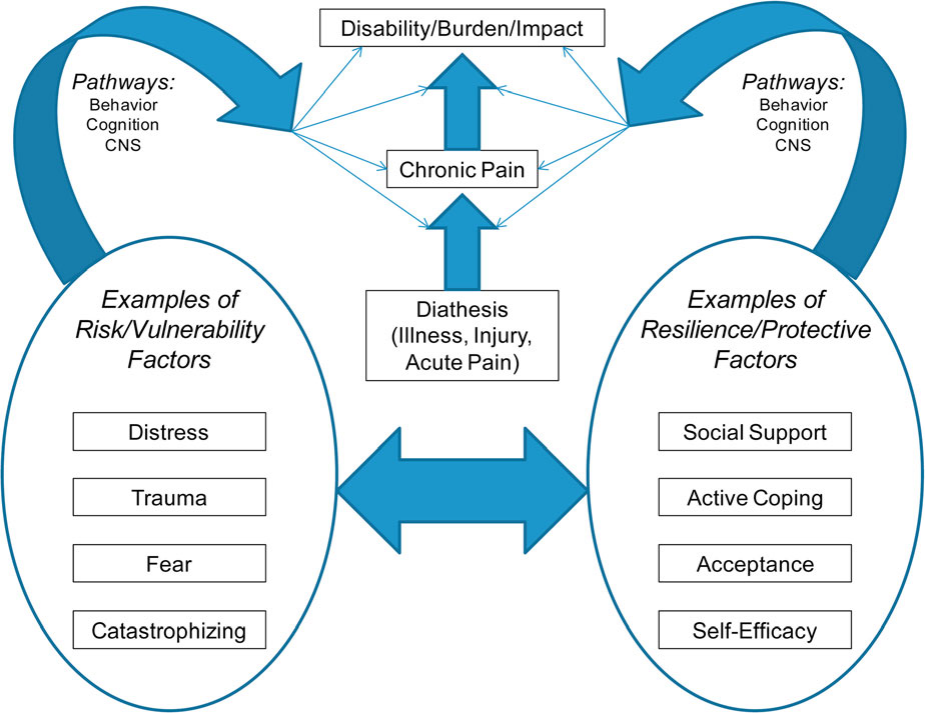
Relationship of psychosocial constructs and processes on pain-related outcomes [29]

**Fig. 2:**
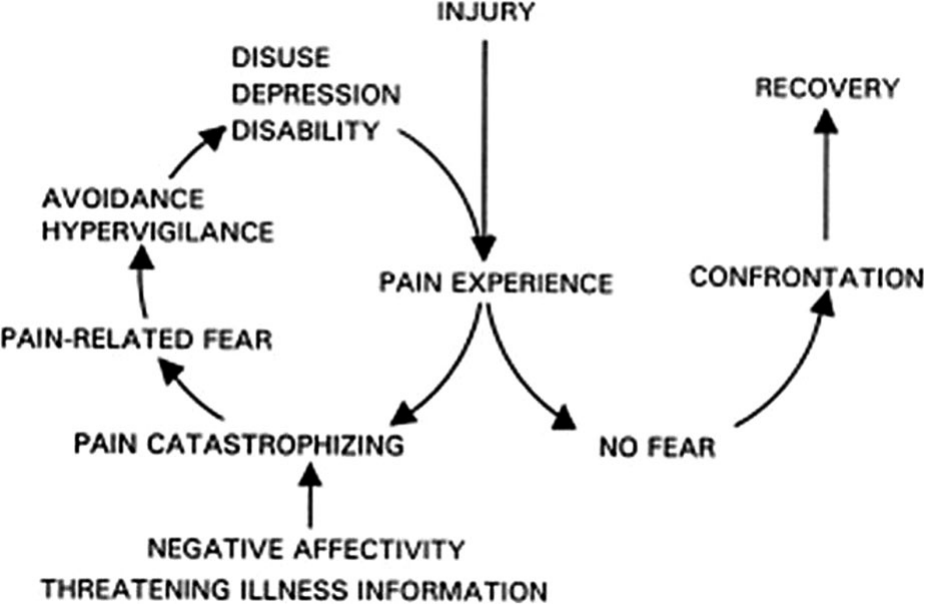
Fear avoidance model of Pain (From: Vlaeyen and Linton.) (Edwards et al. for permissions) [29]

The role of psychosocial factors or yellow flags (anxiety, depression, pain-related catastrophizing, fear-avoidance, self-efficacy for managing pain, and pain-related coping) has been well documented in the development of and maintenance of chronic pain [29–31]. (Fig. 1, Fig. 2).

**Fig. 3:**
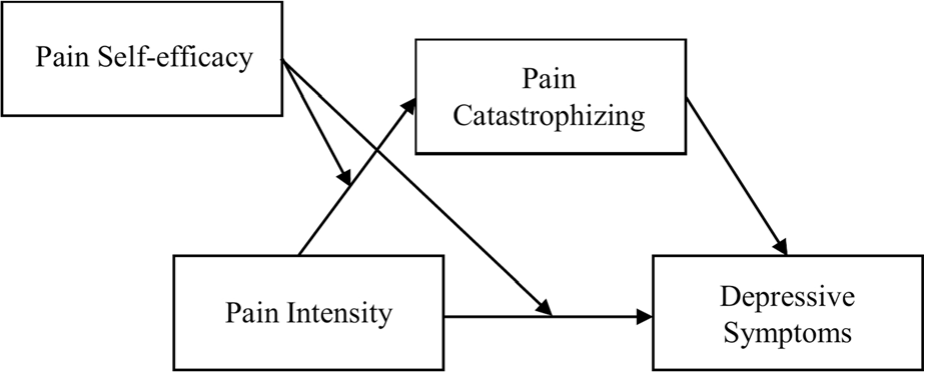
Interrelationships among pain intensity, pain catastrophizing, pain self-efficacy, and depressive symptoms [31]

There is also evidence that chronic pain is a predictor of suicide with chronic pain patients doubling the rate of suicide compared to those without chronic pain conditions [32, 33]. Spinal pain chronicity and suicide risk factors appear to overlap in many domains of the psychosocial profile [29, 32, 34–40]. (Fig. 3).

The chiropractor’s role in primary prevention has been described as a public health approach, with a principal focus on the prevention of musculoskeletal disorders [9, 12]. This valuable, but limited, perception of the chiropractor’s role in public health may have the unintentional consequence of deterring chiropractic physicians from taking initiative in recognizing the patient engaging self-directed violence which may lead to fatal consequences. This is particularly complicated and cumbersome for practitioners working in an outpatient or private practice setting where conversations cannot easily be continued with onsite experts in suicide risk assessment [41]. Of interest, chiropractors have been shown to be able to identify intimate partner violence as portal-of-entry providers [42].

### Suicide prevention is everyone’s business including chiropractors

**Fig. 4:**
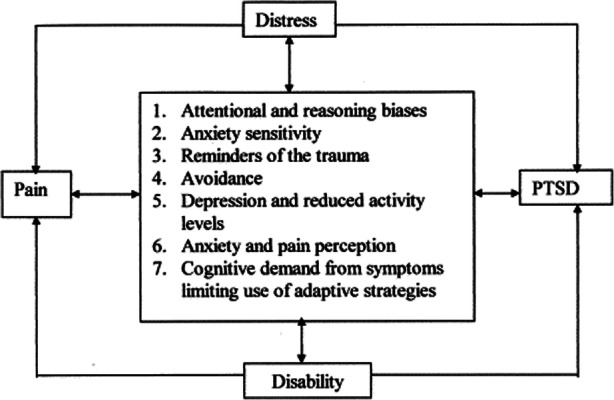
Chronic pain and Post-traumatic Stress Disorder: mutual maintenance [55]

Suicide prevention is every health professional’s concern including chiropractors [43, 44]. The literature specific for chiropractors is scant on the topic of suicide prevention despite former US Surgeon General David Satcher’s effort to initiate social change with the 2001 National Strategy for Suicide Prevention (NSSP 1.0) [45, 46]. Josefowitz reported that 5 out 21 chiropractors had provided care to patients who died by suicide, and 13 of 21 chiropractors had provided care to a patient who had talked about suicide [47]. A longitudinal cohort study of active duty service members with chronic pain exposed to non-pharmacological interventions, including chiropractic care, were at lower risk of new-onset substance use disorder, opioid overdose, suicide ideation, and self-inflicted injuries, including suicide attempts, once enrolled in the Veterans Health Administration [48]. Clinically, patients already present to the chiropractor’s clinic with known risk factors for suicide, the same co-morbidities with relationship to spinal pain that includes, but not limited to, depression, anxiety, and PTSD [28, 49–55]. (Fig. 4) Specific to spinal pain and self-directed violence, including suicidal behavior, the literature is sparse [56–62]. It is our ethical obligation to function at the height of our professional training to serve the public as experts of the musculoskeletal system as well as promoters in each of our communities for public health approaches to health and wellness [63–65].

### An ethical obligation

Given that suicide prevention is a public health issue, it is ethically justifiable to consider chiropractors responsible to recognize risk factors for self-directed violence, including suicide, as active participants of their health care communities. Respect for the patient’s autonomy is not absolute and must be weighed and balanced with beneficence and nonmaleficence [41]. (Table 2).

**Table 2:** Ethics terminology adapted from Sita et al. [41]

Term	Definition
Autonomy	The ability to make one’s own decisions
Beneficence	Doing good on behalf of the patient
Nonmaleficence	Not causing harm to the patient

The chiropractic profession should weigh the public health concern as well as their commitment for health promotion with the overall benefits to the community and the possibility of causing more harm if not addressing suicidality in practice. The principle of justice also requires health care professionals to distribute benefits, risks, costs, and resources. The value of justice in healthcare requires that there is an element of fairness across all practices and would expect chiropractors to uphold a code of ethics as a living document that obligates integrity and accountability in practice. One example is the American Chiropractic Association’s Code of Ethics [66], specifically Tenet X, which states,

“*Doctors of chiropractic should conduct themselves as members of a learned profession and as members of the greater healthcare community dedicated to the promotion of health, the prevention of illness and the alleviation of suffering. As such, doctors of chiropractic should collaborate and cooperate with other health care professionals to protect and enhance the health of the public with the goals of reducing morbidity, increasing functional capacity, increasing the longevity of the U.S. population and reducing health care costs.”*

This ethical obligation suggests the chiropractor assesses and identifies suicide-related risk factors in efforts to achieve the best outcome for patients. It is thus our professional responsibility and social contract to promote public health initiatives that include suicide prevention.

### Public health and the chiropractic profession

Public health initiatives are important components of community health and as chiropractors, we are situated to support these initiatives. Through health education and advocacy, chiropractors are well-suited to support community and individual public health primary prevention needs and to have an influence on policies and education that affects the overall health and well-being of their communities [23]. The potential contributions of chiropractors as it relates to public health are often overlooked or even underutilized. It is important for the chiropractic profession to identify priority areas of focus and clearly articulate plans for engagement in public health initiatives [12].

In 2018, the WFC Public Health Committee developed an agenda in alignment with the World Health Organization (WHO) priorities. This agenda aligns the chiropractic profession with an opportunity to pursue collaborations that will ultimately increase awareness of public health initiatives. The key initiatives include healthy aging; opioid misuse; and women’s, children’s, and adolescent’s health [12]. Additionally, the WHO has created a comprehensive Mental Health Action Plan 2012–2020. This plan includes the implementation of strategies for promotion and prevention in mental health, and to strengthen information systems, evidence, and research for mental health [67]. In the US, *Healthy People 2030* has 3 objectives specific to reduce the suicide rate amongst the US population with special interest focused on the adolescent and lesbian, gay, bisexual, and transgender suicide rates [68]. These priorities can readily be adopted as part of a more comprehensive public health plan within the chiropractic profession. For example, Maiers et al. presented recommendations for creating a more culturally sensitive clinic experience for the transgender community [69].

Additionally, the American Public Health Association (APHA) is the largest public health association in the US. Chiropractors were granted the right to form a group identity within the APHA at the conclusion of 1983 [70]. In 2019, the APHA Chiropractic Health Care section hosted collaborative sessions on the behavioral approach for opioid use disorders and chronic pain, which highlighted the importance of identifying solutions and also risk factors related to opioid use and suicide [71]. Deaths from suicide and opioid overdose fall in the category of deaths of despair, which often can be linked to the breakdown of social determinants of health. The objectives of the sessions were to prepare participants to identify non-pharmacological approaches to pain management and to describe at least two behavioral approaches to addressing opioid use disorder. This collaborative effort culminated in educating attendees on the need to investigate the impact of behavioral and social interventions, including non-pharmacological therapies, designed to improve adherence to medication-based treatment for opioid use disorder and for the management of chronic pain.

### Education and training

The *Council on Chiropractic Education (CCE)* is recognized by the United States Department of Education and the Council for Higher Education Accreditation as the accrediting body for doctor of chiropractic programs (DCPs) and doctor of chiropractic residency programs (DCRPs) [72]. *CCE* meta-competencies cover public health domains concerning mental and behavioral health inclusive of suicide awareness and prevention, and are embedded within meta-competency 3 (Health Promotion and Disease Prevention) of the *CCE* Accreditation Standards DCPs [73]. It is up to the individual institutions to develop curriculum to meet the *CCE* meta-competencies regarding public health and thus suicide awareness which informs the student of their moral and professional responsibilities, medicolegal liabilities, and the practical knowledge necessary to help this urgent patient population.

Although there has been growth in opportunities for training in interdisciplinary settings, there is a shortage of opportunities for chiropractic trainees to learn and train directly with behavioral health professionals [74, 75]. A survey of Australian chiropractors found that only 12% work directly alongside psychologists [76]. Within the US, The Joint Commission, Veterans Affairs, and Department of Defense recommend appropriate training for the management of suicidal ideation in all healthcare settings [77, 78]. There is an opportunity to assess the depth and breadth that which DCPs and DCRPs are providing training related to suicide prevention*.*

### Professional competencies and research

Further, little has been published about suicide awareness for the chiropractic profession to guide professional competency development. Suicide prevention-related recommendations are alluded to by best practice guidelines specific to chiropractic care for older adult population [53] and health care promotion, disease prevention, and wellness [37], with both documents calling for mental health referral when symptoms are recognized. In a chiropractic integrated care pathway for veterans, Lisi et al. recommend reviewing patient records to assess for risk factors of mental health disorders, including a prior suicide attempt [54]. Several books geared towards DCPs include chapters specific to mental health and self-harm [79, 80]. We are aware of only 1 original practice survey study [47] and a related letter to the editor [81] specific to suicide awareness and training for the chiropractic profession in existence.

Challenges and solutions in recognizing and assisting suicidal patients reach far beyond the chiropractic profession. Caine’s public health agenda for suicide prevention identified 5 distinct challenges to preventing suicide [82]. With Challenge 1, there are significant limitations in discriminating against the relatively few true suicide cases from the large number of false-positives. Challenge 2 is the inadequacy to detect false-negatives [83–85]. Challenge 3 is an inability of clinical services to reach many individuals with suicidal intent [86]. Challenge 4 is a paucity of knowledge about fundamental biological, psychological, social, and cultural factors that contribute to apparent risk among diverse populations and groups. Challenge 5 is a lack of coordinated strategies between local, regional, state, and national agencies which would play a role in suicide prevention. Caine called for integrating injury prevention and mental health perspectives to develop public health interventions and address the diversity of populations who contribute to the burdens of suicide and suicide attempt. Additionally, there are individual characteristics, including various assumptions and/or beliefs about suicide, that may serve as barriers to successful assessment of depression or suicide risk.

## Discussion

### Opportunities for suicide prevention in the chiropractic profession

#### Primordial and primary prevention (Fig. 5)

The profession, historically, has demonstrated interest in, and promotion of, public health nationally and internationally, and suicide prevention should be no different. There are numerous successful campaigns and journal articles representative of the chiropractic profession’s collaborative national and global efforts to participate in public health initiatives [64, 87, 88]. Yet, there has been no large professional action to work towards a unified effort for suicide prevention in the primordial or primary prevention stages. It is reasonable to consider individual chiropractors, academic institutions, and professional associations to involve themselves in local, regional, national, and non-profit efforts to promote upstream efforts for suicide prevention. DCPs may introduce evidence-based concepts of suicide prevention into the classroom and through case management vignettes. Chiropractors may engage in effort with the local public health department or partner with national organizations to educate their local community on suicide prevention. Professional trade associations can advocate fostering dissemination of evidence-informed facts to their constituents to enhance clinical acumen and encourage collaboration with other healthcare providers.

**Fig. 5:**
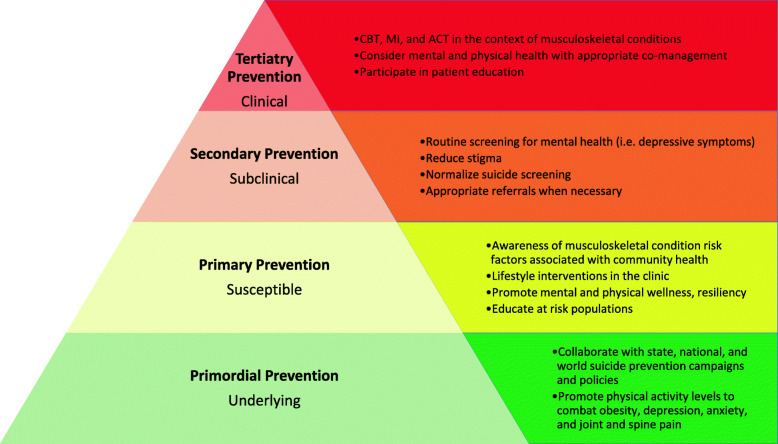
Tiered model of public health suicide prevention with examples for the chiropractic profession

Already integrated into many US Veteran Affairs facilities, chiropractors and chiropractic trainees are uniquely positioned to be part of an inter-agency, collaborative effort to fundamentally change the treatment of mental health and promotion of suicide prevention for not just veterans, but all Americans. To this point, this organization updated evidence-based guidelines in suicide screening and management as recent as 2019 [78]. A recent executive order, the *President’s Roadmap to Empower Veterans and End a National Tragedy of Suicide* (PREVENTS) signed on March 5, 2019, called for the development of a comprehensive national public health roadmap for preventing suicide among American veterans [89]. This not only improves care for veterans, but also ensures critical training experience in suicide prevention to the next generation of all health care trainees, including chiropractic trainees, who are trained through the Veterans Health Administration.

#### Secondary and tertiary prevention

Many will consider our most fitting role in the clinical environment pertains to secondary prevention otherwise known as subclinical detection or screening for suicide and risk factors. Recognition of pronoun misuse and striving to adapt a culturally competent clinic through updates in written and verbal clinic communication can provide a welcoming and safe environment for the transgender community, an at-risk sub-population [69]. Chiropractors should be alert to symptoms of mental health conditions (i.e. depression, anxiety, or PTSD) in patients, especially those with chronic pain, and have an appropriate referral process in place for patients who need a diagnosis and/or treatment of depression or other psychological issues. We should also be cognizant of risk factors and protective factors related to suicide to better identify those individuals who may be more prone to future suicidal ideations. A recent state-of-the-art conference, involving a team of expert clinicians across health care disciplines, recommended considerations for integrated treatments (e.g., for pain and depression) as there may be an additive benefit to treatment approaches that combine modalities (e.g., psychological and exercise) relative to single modality approaches [90]. Concerted efforts to identify individuals in crisis or at risk of crisis allow chiropractors to contribute meaningfully to tertiary prevention in referring these patients swiftly.

With awareness and movement to active considerations for suicide prevention, chiropractors, as portal-of-entry providers, identify depressive symptoms and refer to an appropriate health care provider. Screening for depression increases the number of diagnosed patients, which subsequently allows for the treatment of depression [91]. Further, the patient that shares thoughts of depression or suicide in the chiropractor’s office will do so because of the established therapeutic relationship that is caring, reassuring, sympathetic, and egalitarian [14].

Unknowingly, the chiropractor, may address risk factors and enhance protective factors of suicide during the course of care for a spinal complaint. Frequently, care may address the following: painful musculoskeletal complaint, suffering, disability, hopelessness, helplessness, fear-avoidance, catastrophizing, anxiety, recovery of activity, and return-to-work [32, 38]. Considerations of meaningful relationships, whether in the setting of return-to-work, recovery of activities, and volunteerism, will impact disconnection and isolation, known risk factors concerning suicide. By adapting our clinical environments to consider secondary prevention with suicide risk screening, we not only benefit our patients, but contribute meaningfully within our health care professional community.

#### Research

The profession at large has a profound opportunity to investigate and contribute to the knowledge-base related to suicidal ideation, suicide risk factors (i.e. anxiety, depression, chronic pain), and suicide protective factors (i.e. connectedness, problem solving skills, self-efficacy, etc.). There is a limited understanding related to association of suicide and spinal disorders at the individual and population level. Gaps in the literature exist related to the implementation of secondary prevention strategies into clinical training and clinical practice of chiropractors. Understanding risk factors and protective factors can be exploited to enhance our communication strategies and better understand the chiropractor’s role in suicide prevention by addressing painful musculoskeletal complaints upstream of self-directed violence.

We recommend efforts to delineate the relationship between non-pharmacological treatments for spinal disorders and resultant protective or risk factors relative to self-directed violence. It is prudent to clarify the role of chiropractors in addressing not only secondary prevention strategies, but also primordial, primary, and tertiary suicide prevention strategies. With so little research on the topic at this junction, many possibilities exist related to suicide and spinal disorders.

## Conclusion

To date, we are unaware of any profession or society endorsed measures or recommendations for implementing suicide prevention for the chiropractic profession. We call for the profession to be *EPIC*, and to be *evidenced-based* on the risk factors of suicidal behavior, to be *patient-centered* and listen to our patients, to be *interprofessional* and network with our health care colleagues, and finally to be *collaborativ*e in the best interest of our patients [17].

Taking action involves raising awareness and increasing education for profession-level involvement to promote a public health approach to suicide prevention. Chiropractic clinicians, researchers, and association leaders have a professional responsibility to play a role to address the mental health needs of those with musculoskeletal complaints. The chiropractic profession must act now and be part of the solution to suicide prevention.

## Data Availability

Not applicable.

## Abbreviations

APHA: American Public Health Association
CCE: Council on Chiropractic Education
DCPs: Doctor of chiropractic programs
DCRPs: Doctor of chiropractic residency programs
EPIC: evidence-based, patient-centered, interprofessional, and collaborative
PTSD: Post-traumatic stress disorder
US: United States
WFC: World Federation of Chiropractic
WHO: World Health Organization
